# Mojo Hand, a TALEN design tool for genome editing applications

**DOI:** 10.1186/1471-2105-14-1

**Published:** 2013-01-16

**Authors:** Kevin L Neff, David P Argue, Alvin C Ma, Han B Lee, Karl J Clark, Stephen C Ekker

**Affiliations:** 1 Department of Biochemistry and Molecular Biology, Mayo Clinic College of Medicine, Rochester, MN, USA

**Keywords:** TAL effector, TALEN, Genome editing

## Abstract

**Background:**

Recent studies of transcription activator-like (TAL) effector domains fused to nucleases (TALENs) demonstrate enormous potential for genome editing. Effective design of TALENs requires a combination of selecting appropriate genetic features, finding pairs of binding sites based on a consensus sequence, and, in some cases, identifying endogenous restriction sites for downstream molecular genetic applications.

**Results:**

We present the web-based program Mojo Hand for designing TAL and TALEN constructs for genome editing applications (http://www.talendesign.org). We describe the algorithm and its implementation. The features of Mojo Hand include (1) automatic download of genomic data from the National Center for Biotechnology Information, (2) analysis of any DNA sequence to reveal pairs of binding sites based on a user-defined template, (3) selection of restriction-enzyme recognition sites in the spacer between the TAL monomer binding sites including options for the selection of restriction enzyme suppliers, and (4) output files designed for subsequent TALEN construction using the Golden Gate assembly method.

**Conclusions:**

Mojo Hand enables the rapid identification of TAL binding sites for use in TALEN design. The assembly of TALEN constructs, is also simplified by using the TAL-site prediction program in conjunction with a spreadsheet management aid of reagent concentrations and TALEN formulation. Mojo Hand enables scientists to more rapidly deploy TALENs for genome editing applications.

## Background

TAL domains exhibit programmable, sequence-specific binding to DNA, a feature that makes them a valuable addition to the tools of the molecular biologist. In particular, TAL domains may be used in combination with endonucleases to cause double-strand breaks which are exploited for genome editing, either by error-prone non-homologous end-joining repair of double-strand breaks or insertion of new sequence by homologous recombination. These exciting possibilities depend on the ability of a molecular biologist to design TAL binding sequences for specific genomic regions.

Sequence-specific DNA binding by TAL effectors is accomplished by individual sub-domains of 33–35 amino acids. These repeat variable di-residue (RVD) [1,2] domains contain a central pair of amino acids that determine the base to which it binds. A variety of these RVDs are found in nature, but artificial TAL effectors typically include: adenine=NI, cytosine=HD, guanine=NN, and thymine=NG. For example, these RVDs are used in the one-pot Golden Gate [3], FLASH [4], unit assembly [5], or iterative capped assembly [6] reactions to construct sequence-specific DNA binding proteins.

The TAL domain can bind nearly any DNA sequence. Early work on TAL effectors indicated a consensus sequence where a thymine must precede the binding site, followed by [ACG] and [CGT] [1-4,6]. These requirements are remarkably nonrestrictive, which makes the TAL proteins useful for targeting most genes and regulatory elements of sufficient length. Recent work, however, indicates that only the first of these consensus sequence rules appears to be a measurable constraint on TALEN design [7] (ALM, unpublished results), though even that may not be absolute when using appropriately designed N and C termini [6-8].

In the context of genome editing, software for designing binding sites for TALENs should be flexible and able to target both exons and introns. Also, detecting TALEN activity is often facilitated by restriction fragment length polymorphism (RFLP) assays (for example, [9]). Access to design software in a context suitable for use by molecular biologists and life scientists is also essential for TALEN use by the field.

## Results and discussions

**Figure 1:**
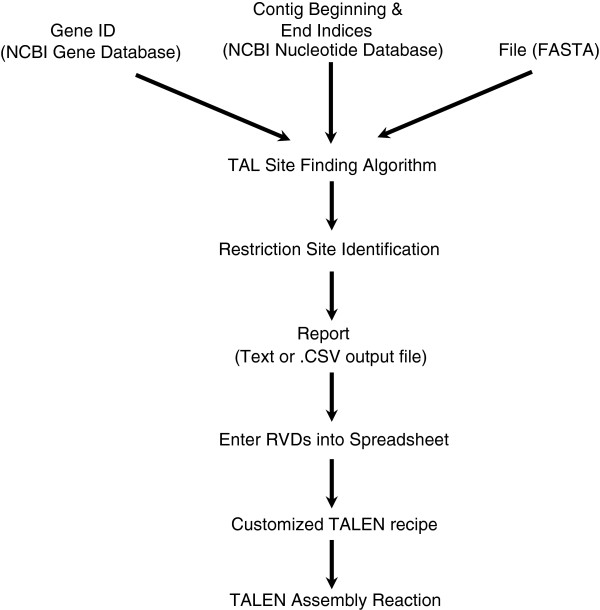
**Flowchart of Mojo Hand**. **Inputs are the Entrez Gene identifier, a nucleotide fragment, or a text file containing the sequence in FASTA format.** The output is a report containing possible binding sites, suitable restriction sites, and RVDs. This output can be further processed with the included spreadsheet, which produces a customized recipe for each TALEN.

The Mojo Hand algorithm consists of multiple steps: user definition of sequence source, retrieval of non-FASTA sequence, identification of candidate TALEN sites, selection of restriction enzyme sites for TALEN efficiency analysis, and output to a text or .csv file. The output can be used directly or pasted into a supplied spreadsheet to ease in assembly of the designed TALENs. The process is depicted in Figure 1.

### Sequence input

Entering the sequence to be targeted by the TAL effector may be accomplished through automatic download of the gene through the NCBI Gene or Nucleotide databases or FASTA-formatted text file. Users specify the unique identifier for the gene of interest. Then the exons and introns of which are retrieved using E-Utilities [10], the API for Pub-Med and other NCBI databases.

To process subsequences (exon or intron) in genes downloaded from NCBI Gene, Mojo Hand requires at least one mRNA, CDS, misc RNA, or exon feature. No subsequence information is used when users enter an arbitrary sequence through FASTA files or Nucleotide requests. The Gene record must also include genomic location information so that the correct genomic sequence may be requested from the NCBI Nucleotide database. In a random sample of genes from diverse organisms, we found that 80% of genes were precisely located. Of these, we noted that 63% had at least one mRNA record, 89% had at least one CDS record, and 4% had at least one miscellaneous RNA record (Table 1). Only 5% of the genes lacked all subsequence features (below). In some cases, features for several genes (other reading frames, especially on the opposite strand) are annotated but Mojo Hand can determine which feature to use based on the gene symbol and its aliases. However, when multiple mRNA features are included for the same gene, there is no obvious way to determine which is most appropriate, so Mojo Hand selects the first feature found unless the user specifies a preference by index (or by transcript identifier). Similarly, because CDS records lack transcript or other unique tags, the user may only specify an index preference. Exon features are the smallest defined feature used as a group. Therefore, no further subsequence selection can be made.

**Table 1:** Frequency of various types of GenBank features

**Freq**	**exon**	**mRNA**	**CDS**	**misc RNA**
0	1978	763	213	1975
1	47	993	1416	71
2	15	188	305	5
3	4	71	94	
4	2	20	9	1
5	3	8	6	
6	1	4	3	
7	1	2	4	
8	1		3	

Frequency of feature count for 2052 randomly selected genes. Gene annotations often do not include mRNA records, but most (95%) have at least one CDS record. Miscellaneous RNA features are relatively rare. Genes may also contain multiple subsequence features that include variations in exon locations, identical reports from multiple authors, or genes in other reading frames. This table only includes genes for the ~80% of genes for which precise genomic location.

Many genes are not fully annotated and may not have annotated mRNA features. Mojo Hand prefers mRNA features, but if none are found, CDS and miscellaneous RNA records may be used. If none of these subsequence features exist, the entire sequence is used. The mRNA record is given the highest priority because it gives the user flexibility in selecting any exon. However, users may also set the priority to CDS records (−−cds-index=1) that do not contain promoter and other regulatory elements that may complicate results.

**Figure 2:**
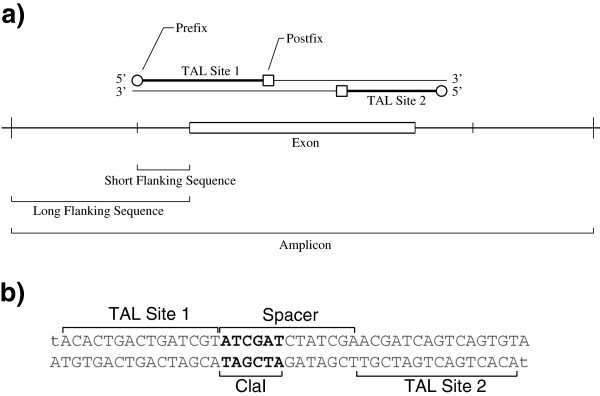
**Schematic of a TALEN and its flanking sequence. a)** An exon with TALEN binding site. The short flanking sequences are needed for processing TAL sites that are near the ends of an exon or for very short exons. The long flanking sequence is used to find enzymes that cut within the amplicon. Binding sites may be defined with a prefix or a postfix. If restriction fragment length polymorphism (RFLP) is used to measure the effect of TALENs, the uniqueness of the enzyme recognition site is found on the span including both long flanking sequences. **b)** Test sequence designed with only one TALEN site. The prefix thymines are indicated with minuscules. The recognition site for the restriction enzyme is in boldface.

Users may select exons or introns for analysis or use the entire sequence. Depending on the input, some flanking sequence may be included for further analysis. The flanking sequence is desirable because some exons are short compared to the length of the TAL binding site. Flanking sequence may also be needed for selecting a restriction enzyme that cuts only once in an amplicon of specific length, especially for binding sites that occur near the ends of an exon or for very short exons as illustrated in Figure 2.

### NCBI access

Sequence data was downloaded using ESummary and EFetch – URL-based methods of requesting information from the various NCBI database. The gene of interest (e.g. gene ID 567858) is requested by the unique identifier in the Gene database first. Mojo Hand then constructs a request for a detailed record based on the genomic location as defined by the RefSeq accession number and indices of the beginning and end of the sequence. The source of these 3 parameters can be automatically determined from the XML output of an ESummary request from a Gene database or manual entry at the command line. For example, Gene 567858 can be requested by Gene Identifier or its location NC 007118.5, 21501610–21527471. This identifier and range define the gene of interest and may be used if the unique identifier in Gene does not produce the desired result. Mojo Hand may also be used to find binding sites in sequences that are not designated as a gene by NCBI. Mojo Hand then requests the detailed record from Nucleotide in XML format using EFetch, captures the beginning and end points of the exons from the mRNA section, and stores the genomic sequence for later analysis. The forward or reverse strand is requested based on the beginning and end indices mentioned above. Because any number of mRNA features may be available, Mojo Hand parses the XML to find those features that are designated with the same symbol as the gene of interest. This procedure distinguishes between the gene of interest and other genes encoded in another reading frame. Some records (e.g. gene ID 32619) have many mRNA entries for the gene of interest. Since there is no automatic way to determine which entry is the most appropriate, the first entry is used. In cases of manual entry of gene location, no symbol is available and the first mRNA is used, and a warning issued. Troublesome cases may be handled by downloading the gene, extracting the exons manually and using the file input mode.

The length of the requested sequence is modified to include a user-defined number of bases upstream and downstream of the gene. If the gene is very near the beginning of the contig, some of the sequence may be undefined and is filled with placeholders. Likewise, if the requested sequence falls near the end of the contig such that the trailing flanking sequence extends beyond its end (e.g. gene ID 802118), placeholders are added.

Records containing long sequences (e.g. gene ID 19091, 1.2 million base pair (bp)) are processed somewhat differently. The XML output is not processed beyond the end of the GSeq feature table field.

### Identification of binding sites

We designed Mojo Hand to identify TAL binding sites based on a user-defined template. We initially used the template sequence of Ts[ACG][CGT].*Te. The notation s and e indicate the start and end of the binding sequence, bases enclosed in brackets represent a choice, and .* indicate zero or more bases for which there is no preference. So this template will identity TAL binding sites that start with a T, are followed by A, C, or G (not a T), and are followed by a C, G or T (not an A). Another template, based on the work of Sun and coworkers [7], is Ts.*e. In practice, we have found no substantial functional constraints besides the initial 5’ T bp, so we now use this as the default template sequence parameter for Mojo Hand.

In addition to the constraints described above, the user specifies a range of allowed lengths for the binding sites and the spacer. The algorithm generates the entire set of possible binding sites based on these lengths. Each candidate binding site is then filtered based on TAL-site restrictions. The candidate sites are made by iterating the possible lengths (Figure 2a) through the user-defined ranges for binding site and spacer lengths. Default values are provided: 15–17 bp for the binding sites, 15–16 bp for the spacer.

When the length of an exon is long, execution time may be extensive and the number of TALEN sites becomes unusably large. Therefore, for long exons, some binding sites are skipped by incrementing the beginning of the first TAL site by values larger than unity. For exons longer than 1000 bp, the increment is 10; for exons longer than 5000, the increment is 20. Using this skipping method and the classical consensus sequence, we observed that many binding sites were found in our test set (35 genes, listed below). Every possible binding site may be obtained by submitting fragments of length 999 base pairs or less as a FASTA file or NCBI Nucleotide request.

### Restriction enzyme analysis

The biological effect of TALEN activity may be observed in several ways, including restriction fragment length polymorphism (RFLP), sequencing, and phenotype. If RFLP will be used as an evaluation approach, a restriction site should be present within the space between the TAL binding sites. In conjunction with double-strand break repair by error-prone non-homologous end-joining (NHEJ), the nearby restriction site is often disrupted. We subjected candidate binding site pairs and spacers to further analysis to find those candidates with unique restriction-enzyme binding sites within their spacer region. If the user requests the single TAL binding site option, no enzyme analysis is performed.

We used REBASE [11], a database of restriction enzymes hosted by New England Biolabs (NEB), as the default restriction enzyme database collection within Mojo Hand. We use only the subset of enzymes that are commercially available. Future releases of the REBASE database may be downloaded and used in the place of the version distributed with this manuscript. Custom in-house databases may also be used if the format matches that of REBASE. We also used results published by NEB [12] to determine which enzymes were compatible with several PCR buffers (standard, Thermopol, Phusion, and Crimson). These scores are rescaled on the range [0,9] and displayed with each enzyme. Formal and prototype names (first described enzyme of a particular family) are displayed for ease of use. Mojo Hand permits selection of enzyme site screening by vendor(s). Narrowing the selection decreases computational load thereby decreasing processing time for TALEN site selection using Mojo Hand.

### Mojo Hand output

**Figure 3:**
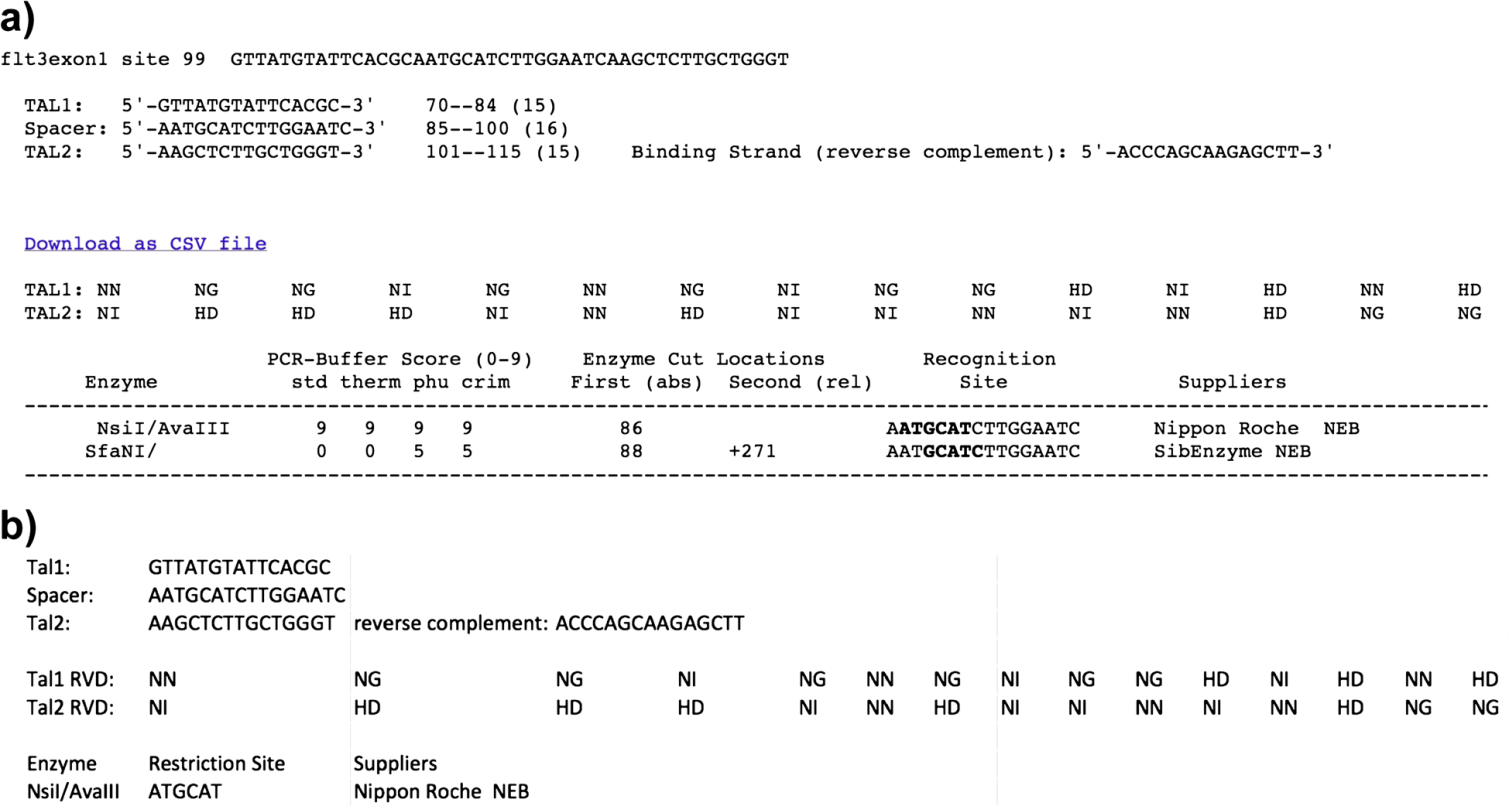
**Example output of Mojo Hand. a)** Screen-shot of output from Mojo Hand for zebrafish gene flt3 Exon 1. Of all possible TALEN binding sites, this is Site 99. The record includes the binding sequences in proper orientation, the RVDs (tab delimited in normal output for easy transfer to a spreadsheet) and the restriction enzymes that could be used. Note that this example has only a single cutting site so the absolute position of the beginning of the restriction site is given but the column for relative position of the second cut is empty. **b)** Screen-shot of downloaded spreadsheet in .CSV format of the same example.

The output of Mojo Hand is either a flat text file or HTML that includes the sequences to which the TAL proteins will eventually bind, RVDs, and enzymes that cut in each spacer (Figure 3a) or a downloadable comma-separated values (.csv) file that can be imported into common programs like Microsoft Excel (Figure 3b). The binding sites are denoted TAL1 and TAL2, as illustrated in Figure 2b. The first site and spacer are considered the forward direction, regardless of the strand in the NCBI Nucleotide database. The reverse complement of the second binding site is also provided to show the nucleotide sequence to which the TALEN protein will bind.

The gene symbol is indicated at the beginning of each TALEN site. The notation symbol E# indicates which exon each binding site was found within. The prefix E indicates that the region of interest was an exon; I for intron, and A when no subsequence region was available and the entire sequence was used.

The position and length of each binding site are provided. These values are relative to the beginning of the subsequence fragment (exon or intron) and include the user-defined short flanking sequence. The notation #–#(#) indicates the first and last index of the binding site, with the length in parentheses.

The restriction sites for RFLP are listed for each TALEN site. The enzyme name is listed with its prototype. The compatibility of the enzymes with full-strength PCR buffer [12] is listed, rescaled so that it falls on the range [0,9], with 0 representing no activity. The position of the first base of the enzyme’s recognition sequence is given for nucleases that cut only once in the amplicon (binding site with default of 150 flanking bp on either end). The minimum distance to the second cut site is configurable (default = 80 bp). If an enzyme cuts in two positions that are at least this minimum distance apart, the second cut is given relative to the first restriction site. The indexing is based on the beginning of the subsequence fragment, including the long flanking length. If using the Mojo Hand web service, enzyme restriction site matches are highlighted in black in the spacer while reverse complement restriction site matches are highlighted in red.

### TALEN recipe generator

Commonly used TALEN assembly protocols involve a large number of plasmids [3]. Mojo Hand includes a spreadsheet that aids in TALEN formulation that may be used in conjunction with the Golden Gate method and the TALEN DNA kit from Addgene (TALEN Kit #1000000016; Cambridge, MA, USA)[3,9,13]. Mojo Hand outputs tab-delimited or CSV downloadable RVD sequences, which can be transferred into the spreadsheet using the clipboard. The spreadsheet then produces recipes that facilitate the molecular assembly of TALENs.

### Verification of Mojo Hand and measurement of TALEN activity

**Figure 4:**
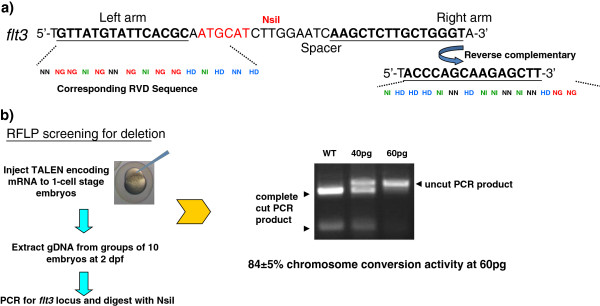
***In vivo *activity of Mojo Hand-designed TALEN. a)** Detail design of TALEN binding site targeting zebrafish flt3 Exon 1 sequence. **b)** Description and results of the RFLP assay showing targeted small deletion (loss of NsiI recognition sequence) introduced by the Mojo Hand-designed TALEN pair. Primers for RFLP assay: Forward-TGAAAGTCTTCTTGCCTCTGTTC; Reverse- CAGCTGTAAATGAGTCTCACAGTT.

To prospectively test the Mojo Hand software, we designed TALENs targeting zebrafish flt3 sequence using Mojo Hand. A 450 bp genomic fragment including Exon 1 was used as input. An Exon 1 targeting TALEN pair with 15-RVD binding sites including a 5’ upstream T nucleotide at both arms was chosen, which has a 16 bp spacer and a unique NsiI site in the spacer (Figures 3 and 4). The TALEN constructs were subsequently synthesized with the Golden Gate Method [3] using the GoldyTALEN scaffold [9,13]. *In vitro* transcribed mRNAs (mMessage mMachine T3 kit, Life Technology, Carlsbad, CA, US) encoding the TALEN pair were injected into the cytoplasm of 1-cell stage wild-type zebrafish. At 48 hours-post-fertilization (hpf), genomic DNA was extracted from single and groups of 10 phenotypically normal embryos. The *in vivo* activity of the TALEN was screened by RFLP assay as previously described [9]. In embryos injected with the TALEN mRNA, uncut PCR product was detected representing the loss of NsiI site through TALEN-induced small deletion, with the percentage of converted chromosomes measured to be 84 ± 5% (average of three different experiments). These results demonstrated a successful TALEN design with Mojo Hand. We have conducted a separate prospective analysis of 12 more TALENs made using Mojo Hand, with each pair garnering over 20% activity and the set averaging over 50% converted chromosomes in this zebrafish embryo assay (data not shown).

### Source code and Web service

Mojo Hand is available as a web service at http://www.talendesign.org. The site allows access to the program without the trouble of installation and with the ease of a familiar interface. Point-of-use help is available for each field. The source code and spreadsheet are also available for non-commercial use with applicable license.

## Conclusions

We developed Mojo Hand based on an initial training set of 35 genes, further tested the program with users from 3 separate laboratories, and finally conducted a prospective study of over a dozen TALEN pairs. During the initial vetting process, we showed that the correct sequences were downloaded from NCBI databases using NCBI nr/ntBLAST [14]. We manually confirmed that the Mojo Hand predicted binding sites were within the expected exon of the correct gene in all cases. We also compared Mojo Hand output to manually retrieved GenBank records and verified several binding sites to ensure that they occurred in the correct exon and that the prefix requirements (a 5’ thymine, in most cases) were satisfied. Our test set was constructed so that different numbers (0–3) of subsequence features were present, allowing us to assess how Mojo Hand prioritizes mRNA, CDS, and misc RNA features. We included genes with multiple aliases and features labeled with an alias to test if all appropriate subsequence records were found.

Mojo Hand complements previously described software such as the ISU TALEN Targeter [3,15] because Mojo Hand addresses the difficulty of downloading the sequence and extracting exons (or introns) based on annotation from GenBank. The ISU TALEN Targeter currently only accepts sequences entered in a text box or FASTA file upload. Mojo Hand can also screen for possible TALEN sites using more extensive databases of restriction enzymes (REBASE) rather than the NEB database that was recently added to the ISU tool program. Mojo Hand also provides a spreadsheet that bridges the gap between the RVD output and the bench. The spreadsheet produces recipes for individual TALENs that take into account local reagent concentrations.

We also compared our work to idTALE, a web service provided by King Abdullah University of Science and Technology [16]. idTALE allows users to provide sequence directly or by Ensembl gene identifier. Genes are, however, restricted to just a few species (A. thaliana, P. patens, C. elegans, D. melanogaster, S. cerevisiae), and no restriction analysis is done. Mojo Hand appears more flexible because any gene or sequence can be entered, any consensus sequence may be used, and restriction analysis is available.

Beyond the single RVD to nucleotide recognition cipher of TALENs, other interactions that affect TALEN activity appear to be minor. However, factors that potentially affect TALEN efficiency continue to be investigated. New information regarding TALEN design may require rapid change of current TALEN design software. Therefore, Mojo Hand has been designed to permit user-defined adjustments. Beyond TALEN design based on binding alone, Mojo Hand provides an integrated a way to download the exons or introns of any gene and to filter the results based on restriction enzyme recognition sites.

We have designed Mojo Hand to be as general as possible, but there are several limitations. The annotated features in GenBank can vary based on what is known about a particular gene, so Mojo Hand may not be able to download certain predicted genes. We found that a significant proportion of randomly selected genes in NCBI have no subsequence records (5%, Table 1), and many (20%) have no genomic location. Also, we limit our automatic download features to work only on genes in the GenBank and NCBI Nucleotide databases, which excludes much of the non-coding, non-repetitive regions of the genome. Users may manually enter sequence data to overcome this limitation.

## Availability and requirements

The web-based interface of Mojo Hand is designed for ease of use, and the source code is available for non-commercial use through an applicable license to enable programmatic interface development by advanced users. These multiple interfaces to this flexible software tool are designed to empower researchers to exploit TALENs for genome editing applications.

## Abbreviations

TAL: Transcription activator-like; TALEN: Transcription activator-like effector nuclease; NCBI: National Center for Biotechnology Information.

## Competing interests

The authors declare that they have no competing interests.

## Authors’ contributions

KLN designed and implemented the initial program Mojo Hand and co-wrote this manuscript. DPA implemented the CGI-based web site and further developed and tested the Mojo Hand program. KJC worked with KLN and DPA to define user needs and the biological requirements of the TALEN design process, designed the TALEN assembly spreadsheet tool, and co-wrote the manuscript. ACM and HBL constructed the flt3 TALEN; ACM analyzed the flt3 TALEN efficiency in zebrafish, and co-wrote the manuscript. SCE provided scientific project oversight and co-wrote the manuscript. All authors read and approved the final manuscript.

## References

[B1] Moscou MJ Bogdanove AJ A simple cipher governs DNA recognition by TAL effectors Science 2009 326 1501 1501 10.1126/science.1178817 19933106

[B2] Boch J Scholze H Schornack S Landgraf A Hahn S Kay S Lahaye T Nickstadt A Bonas U Breaking the code of DNA binding specificity of TAL-type III effectors Science 2009 326 1509 1512 10.1126/science.1178811 19933107

[B3] Cermak T Doyle EL Christian M Wang L Zhang Y Schmidt C Baller JA Somia NV Bogdanove AJ Voytas DF Efficient design and assembly of custom TALEN and other TAL effector-based constructs for DNA targeting Nucleic Acids Res 2011 39 e82 10.1093/nar/gkr218 21493687 PMC3130291

[B4] Reyon D Tsai SQ Khayter C Foden JA Sander JD Joung JK FLASH assembly of TALENs for high-throughput genome editing Nat Biotechnol 2012 30 460 465 10.1038/nbt.2170 22484455 PMC3558947

[B5] Huang P Xiao A Zhou M Zhu Z Lin S Zhang B Heritable gene targeting in zebrafish using customized TALENs Nat Biotechnol 2011 29 699 700 10.1038/nbt.1939 21822242

[B6] Briggs AW Rios X Chari R Yang L Zhang F Mali P Church GM Iterative capped assembly: rapid and scalable synthesis of repeat-module DNA such as TAL effectors from individual monomers Nucleic Acids Res 2012 40 e117 10.1093/nar/gks624 22740649 PMC3424587

[B7] Sun N Liang J Abil Z Zhao H Optimized TAL effector nucleases (TALENs) for use in treatment of sickle cell disease Mol Biosyst 2012 8 1255 10.1039/c2mb05461b 22301904

[B8] Miller JC Tan S Qiao G Barlow KA Wang J Xia DF Meng X Paschon DE Leung E Hinkley SJ Dulay GP Hua KL Ankoudinova I Cost GJ Urnov FD Zhang HS Holmes MC Zhang L Gregory PD Rebar EJ A TALE nuclease architecture for efficient genome editing Nat Biotechnol 2011 29 143 148 10.1038/nbt.1755 21179091

[B9] Bedell VM Wang Y Campbell JM Poshusta TL Starker CG Krug RG Tan W Penheiter SG Ma AC Leung AYH Fahrenkrug SC Carlson DF Voytas DF Clark KJ Essner JJ Ekker SC In vivo genome editing using a high-efficiency TALEN system Nature 2012 491 114 118 10.1038/nature11537 23000899 PMC3491146

[B10] Entrez programming utilities help http://www.ncbi.nlm.nih.gov/books/NBK25501/

[B11] REBASE, the restriction enzyme databasehttp://rebase.neb.com

[B12] Activity of Restriction Enzymes in a Taq or Phusion PCR Mix http://www.neb.com/nebecomm/tech_reference/restriction_enzymes/activity_in_taqPCRmix.asp#top

[B13] Carlson DF Tan W Lillico SG Stverakova D Proudfoot C Christian M Voytas DF Long CR Whitelaw CBA Fahrenkrug SC Efficient TALEN-mediated gene knockout in livestock Proc Natl Acad Sci USA 2012 109 17382 17387 10.1073/pnas.1211446109 23027955 PMC3491456

[B14] Altschul SF Gish W Miller W Myers EW Lipman DJ Basic local alignment search tool J Mol Biol 1990 215 403 410 2231712 10.1016/S0022-2836(05)80360-2

[B15] Doyle EL Booher NJ Standage DS Voytas DF Brendel VP Vandyk JK Bogdanove AJ TAL effector-nucleotide targeter (TALE-NT) 2.0: tools for TAL effector design and target prediction Nucleic Acids Res 2012 40 W117 W122 10.1093/nar/gks608 22693217 PMC3394250

[B16] Li L Piatek MJ Atef A Piatek A Wibowo A Fang X Sabir JSM Zhu J-K Mahfouz MM Rapid and highly efficient construction of TALE-based transcriptional regulators and nucleases for genome modification Plant Mol Biol 2012 78 407 416 10.1007/s11103-012-9875-4 22271303 PMC3580834

